# Serial Plasma Choline Measurements after Cardiac Arrest in Patients Undergoing Mild Therapeutic Hypothermia: A Prospective Observational Pilot Trial

**DOI:** 10.1371/journal.pone.0076720

**Published:** 2013-09-30

**Authors:** Christian Storm, Oliver Danne, Per Magne Ueland, Christoph Leithner, Dietrich Hasper, Tim Schroeder

**Affiliations:** Department of Intensive Care Medicine and Nephrology, Charité Universitätsmedizin Berlin, Campus Virchow-Klinikum, Berlin, Germany; Department of Emergency Medicine, Charité Universitätsmedizin Berlin, Campus Virchow-Klinikum, Berlin, Germany; Section for Pharmacology, Institute of Medicine, University of Bergen and Laboratory of Clinical Biochemistry, Haukeland University Hospital, Bergen, Norway; Department of Neurology, Charité Universitätsmedizin Berlin, Campus Virchow-Klinikum, Berlin, Germany; Tokai University, Japan

## Abstract

**Objective:**

Choline is related to phospholipid metabolism and is a marker for global ischaemia with a small reference range in healthy volunteers. The aim of our study was to characterize the early kinetics of plasma free choline in patients after cardiac arrest. Additionally, we investigated the potential of plasma free choline to predict neurological outcome.

**Methods:**

Twenty patients admitted to our medical intensive care unit were included in this prospective, observational trial. All patients were enrolled between May 2010 and May 2011. They received post cardiac arrest treatment including mild therapeutic hypothermia which was initiated with a combination of cold fluid and a feedback surface cooling device according to current guidelines. Sixteen blood samples per patient were analysed for plasma free choline levels within the first week after resuscitation. Choline was detected by liquid chromatography-tandem mass spectrometry.

**Results:**

Most patients showed elevated choline levels on admission (median 14.8 µmol/L; interquartile range; IQR 9.9-20.1) which subsequently decreased. 48 hours after cardiac arrest choline levels in all patients reached subnormal levels at a median of 4.0 µmol/L (IQR 3-4.9; p = 0.001). Subsequently, choline levels normalized within seven days. There was no significant difference in choline levels when groups were analyzed in relation to neurological outcome.

**Conclusions:**

Our data indicate a choline deficiency in the early postresucitation phase. This could potentially result in impaired cell membrane recovery. The detailed characterization of the early choline time course may aid in planning of choline supplementation trials. In a limited number of patients, choline was not promising as a biomarker for outcome prediction.

## Introduction

The use of mild therapeutic hypothermia has become standard of care for patients after cardiac arrest in order to improve neurological outcome. While many nonspecific details of intensive care for cardiac arrest patients may have changed over time contributing to higher overall survival of patients with initially successful resuscitation, further specific treatments for hypoxic encephalopathy are lacking.

Choline is related to phospholipid metabolism and has been investigated as a marker for global tissue ischaemia.[1]–[4] There is evidence that phospholipase A2 releases choline during membrane breakdown after hypoxia.[2] The supplementation of citicoline as precursor of choline has been investagted as neuroprotective and neuro-regenerative treatment after ischemic stroke. A meta-analysis suggests a benefit but a recent clinical trial could not confirm a positive effect in stroke patients. [5], [6] In patients after cardiac arrest, the whole brain has suffered from a relatively short time of ischemia as compared to ischemic stroke. Thus, neuro-regenerative effects of choline could in principle be more pronounced in cardiac arrest patients as compared to ischemic stroke patients. Reference plasma free choline levels in healthy volunteers have been described in several studies.[7], [8] The time course of plasma free choline levels in patients after cardiac arrest, however, has not been determined in detail yet. In this pilot trial, serial plasma free choline (PLCHO) levels in 20 patients after cardiac arrest undergoing mild therapeutic hypothermia were analyzed to characterize the temporal profile of choline levels early after cardiac arrest.

Furthermore recommended hypothermia treatment may interfere with the validity of several neurological outcome prognostication parameters and additional novel parameters may increase the validity of outcome prediction in a multiparameter apporach.[1]–[4], [9], [10] Several biomarkers of brain cell damage such as protein S-100 and neuron-specific enolase (NSE) have been evaluated and are used for outcome prediction, with NSE cut-off levels substantially higher in hypothermia treated patients.[9]–[11].

In our study with a limited number of patients, we therefore included an analysis of plasma free choline levels as a potential novel biomarker for outcome prediction.

## Materials and Methods

The study protocol was approved by the local ethics committee on human research of the Charité University Hospital and was conducted in accordance with the guidelines of the Declaration of Helsinki. For all patients a healthcare proxy was assigned by a court order to give written informed consent as all cardiac arrest survivors were unconscious on admission. The local ethics committee approved including patients on admission before the healthcare proxy was available. All patients received mild therapeutic hypothermia irrespective of their initial cardiac rhythm according to current guidelines and local standard operating procedure. Mild therapeutic hypothermia was initiated immediately after admission with an intravenous infusion of cold saline (4°C, 1000–1500 ml bolus) followed by surface cooling with a commercially available, non-invasive, computer controlled cooling device (ArcticSun2000® C.R.BARD, Colorado USA). The target temperature of 33°C was maintained for 24 hours, followed by a controlled rewarming procedure with 0.25°C/hour.

**Figure 1:**
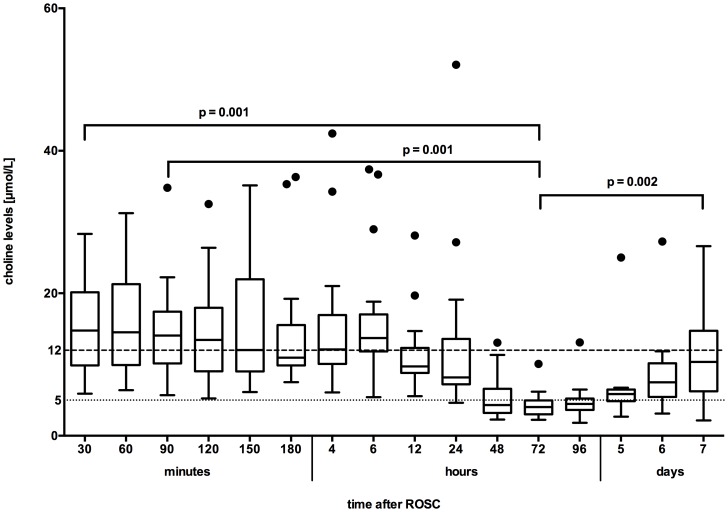
Plasma free choline concentration throughout the study. Dotted line: lower limit reference range; dashed line: upper limit reference range.

Twenty patients were enrolled in this study between May 2010 and May 2011 and blood was taken serially at sixteen different time points. EDTA blood collection tubes (BD PPT™; BD, NJ, USA) were used and all samples were centrifuged at 2.016× g for 10 minutes and then stored at −80°C in order to guarantee sample stability. PLCHO levels were analyzed as defined in our study protocol (see also Figure 1 for time points). During the first 24 hours, samples were collected in short time intervals due to choline's short half-life of approximately 10–25 minutes, furthermore we expected major changes during the first hours after return of spontaneous circulation (ROSC) during hypothermia treatment.[12] We used liquid chromatography-tandem mass spectrometry to determine the PLCHO levels as previously described.[13] The measurements were carried out at the laboratory of Bevital (www.bevital.no; Bergen, Norway). In six patients, plasma free choline levels were not determined at 30 and 90 minutes after ROSC, because percutaneous catheter intervention was performed at these time points. Therefore, from a total of 320 samples (16 time points, 20 patients), 6 samples are missing (2 at 30 minutes and 4 at 90 minutes after ROSC).

The software Statistical Package for the Social Sciences (SPSS version 20, SPSS Inc.; Chicago, Illinois USA) was used for statistical analysis. Since data were not normally distributed, we performed descriptive statistics by using medians and interquartile ranges (IQR) and non-parametric statistical tests (Mann-Whitney U Test, Wilcoxon signed-rank test). Normal distributed numbers have been tested with Pearson test for correlation. GraphPad Prism, version 6.0 for mac (GraphPad Software; La Jolla, California USA) was used to design the graphs.

## Results

Baseline characteristic of the study population are given in Table 1. There was no significant difference concerning time to ROSC (median 22.5, IQR 15.25–40 minutes; p = 0.67) as well as for dosage of adrenaline (epinephrine) (p = 0.43) between the outcome groups.

**Table 1:** Baseline characteristics.

Variable	good outcome	poor outcome	p-value
	(CPC 1–2)	(CPC 3–5)	
	n = 8	n = 12	
Age (years), *median (IQR)*	60.5 (52.7–76)	69.5 (43–73.7)	0.847
Female Sex, *n (%)*	1 (12.5)	5 (41.6)	0.174
APACHE II Score, *median (IQR)*	30 (22.7–39.5)	27 (19–32)	0.263
Out of hospital cardiac arrest, *n (%)*	6 (75)	10 (83.3)	0.656
Shockable rhythm, *n (%)*	7 (87.5)	2 (16.7)	0.002
Witnessed arrest, *n (%)*	7 (87.5)	7 (58.3)	0.174
Bystander CPR, *n (%)*	7 (87.5)	4 (33.3)	0.02
Time to ROSC (min), *median (IQR)*	22.5 (9.7–39)	23 (15.2–40)	0.670
Total adrenaline dose (mg), *median (IQR)*	4 (1.6–5.5)	4.5 (3.2–5.7)	0.433
NSE day 3 µg/L), *median (IQR)*	23 (19–54.5)	164.4 (68–254)	0.003
Time on ventilator (hours), *median (IQR)*	273 (191.5–598.5)	194.5 (136.2–335.7)	0.247
Length of stay (days), *median (IQR)*	21.5 (10.2–36.5)	9.5 (5.5–25.5)	0.177

APACHE =  acute physiology and chronic health evaluation; CPR =  cardiopulmonary resuscitation; CPC =  cerebral performance category scale; NSE =  neuron specific enolase; ROSC =  return of spontaneous circulation

### Choline after cardiac arrest

The reference laboratory in Norway (Bevital; Bergen, Norway) specifies the reference median level to be 8 µmol/L (5^th^–95^th^ percentile 5–12 µmol/L).[7] Most patients showed elevated choline levels on admission (Figure 1). Median choline levels on admission were at 14.8 µmol/L (IQR 9.9–20.1) and started to decrease after six to twelve hours in almost all patients. After 48 hours they reached subnormal concentrations with a median of 4.0 µmol/L (IQR 3–4.9). The decrease was significant (p = 0.001) compared to the baseline level on admission. However, after 96 hours choline levels increased again and reached normal concentrations over the next days of the study period (p = 0.002).

**Figure 2:**
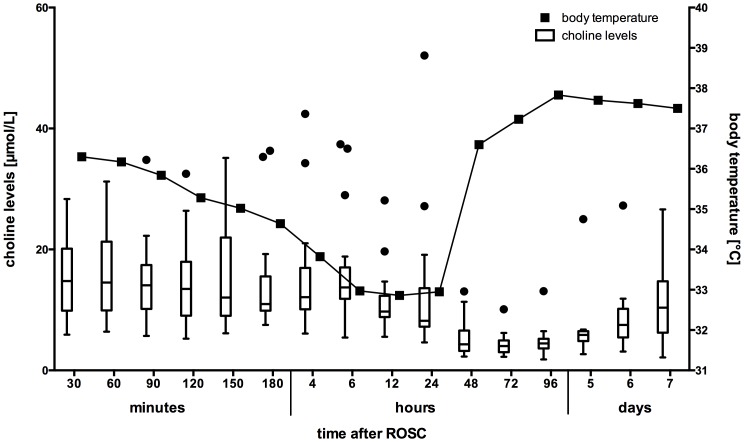
Changes of plasma free choline levels (median and interquartile range) in relation to the body temperature (median).

The target temperature of 33°C during mild therapeutic hypothermia treatment was reached in all patients and rewarming was initiated after 24 hours of maintenance. Of note, all patients had normal temperature at the time of lowest choline levels 72 hours after ROSC and interestingly PLCHO levels remained low for further 48 hours after return to normal body temperature (Figure 1 and Figure 2). The overall lowest choline concentrations were measured 48–72 hours after ROSC. There was no significant correlation between choline levels and body temperature at any time point (p>0.05).

### Choline levels and outcome

**Figure 3:**
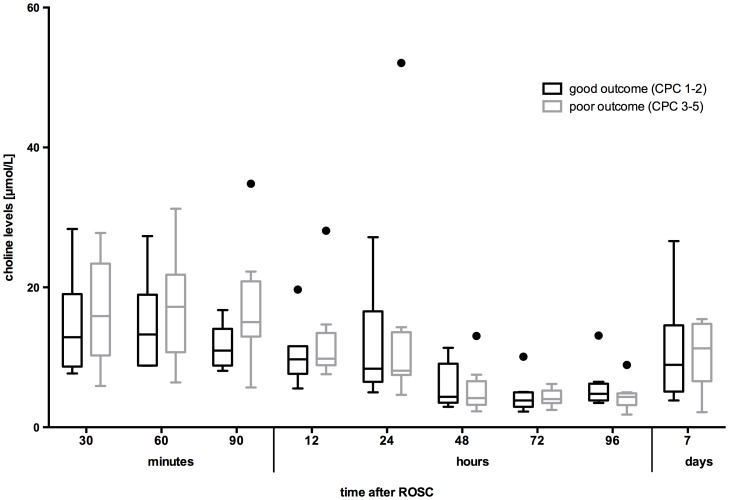
Plasma free choline concentration throughout the study in both subgroups.

Overall 40% of the study population (8/20 patients) achieved good outcome (cerebral performance category; CPC 1–2), and 40% of the patients died (CPC 5). For further outcome analyses patients were subdivided into two groups according to neurological outcome (group 1: good recovery, CPC 1–2, n = 8; group 2: poor outcome, CPC 3–5, n = 12). Patients with poor outcome (group 2) showed slightly higher PLCHO levels on admission, 15.9 µmol/L (10.3–23.4) as compared to 12.9 µmol/L (8.7–19) in the good outcome group, without significance (p = 0.75) (Figure 3). At 90 minutes after ROSC 8 out of 11 patients with poor outcome (group 2, n = 12, one missing value) showed choline concentrations >13 µmol/L whereas in the group with good outcome 4 out of 5 patients (group 1, n = 8, three missing values) remained below this value. At 90 minutes after ROSC, 4 out of 11 patients with poor outcome had choline levels above 17.2 µmol/L, but none of 5 patients with good outcome. In this limited patient cohort, however, we noted no significant difference in plasma choline levels, and no patient with dramatic choline increases which might have suggested a potential for outcome prediction and determination of a cut-off level.

## Discussion

This is the first study characterizing the pattern of plasma choline in hypothermia treated patients after cardiac arrest over seven days, undergoing 24 hours of hypothermia treatment at 33°C. Serial choline levels changed significantly with respect to the reference range. Our main finding is an increase of plasma choline levels shortly after resuscitation followed by a sustained and profound decrease between 48 hours and five days after cardiac arrest. Our data based on a limited number of patients do not indicate a revelant potential of choline levels for outcome prediction.

Previous trials described a release of choline from hypoxic tissue into the plasma and therefore elevated PLCHO levels.[1], [2] The extent of choline release may reflect the severity of global tissue damage due to hypoxia. In our study most patients showed elevated PLCHO levels on admission after successful resuscitation. Patients with poor outcome showed slightly higher levels, but the difference was not statistically significant. The use of PLCHO for risk stratification in patients with acute coronary syndrome has been described before.[14]–[16] In an animal model moderate global hypoxia resulted in an increase of choline in the blood.[2] However, in our study with a limited number of patients after cardiac arrest the difference between outcome groups was relatively small and did not indicate a relevant potential for poor outcome prognostication with high specificity.

In contrast to the initial choline levels within the first hours after cardiac arrest, a significant decrease of choline concentrations to low or even subnormal concentrations starting six to twelve hours after ROSC and affecting all patients was observed. Whether the decline of choline levels in the rewarming phase is coincidental or pathophysiological related remains unknown. There was no significant correlation between choline levels and body temperature at any time point.

However the increased perfusion and metabolism of previously cooled tissue during this phase provides a plausible explanation for a mismatch between an increased tissue demand for choline and choline supply leading to an profound decrease of plasma choline at this time. A decrease of choline levels in acute disease, injury and surgery has been described previously.[17] Metabolic effects of the neurohumoral stress response have been suggested as a potential mechanism for this decline. Figure 3 shows that choline levels further decline as the patient is rewarmed after hypothermia.

In our study a profound decrease of choline levels in patients below the fifth percentile of the reference range (<5 µmol/L) was found. A decrease of serum choline from initial values of 12.3±0.5, 12.1±0.4 and 11.4±0.4 µmol/L has been described for patients undergoing surgery (hysterectomy, off-pump coronary artery grafting surgery and brain tumor surgery) with a mean level of 7.9 µmol/L as the lowest value.[17] Ulus et al. showed a choline decrease to 7.1±0.9 µmol/L two days after surgery in patients undergoing major surgical intervention or childbirth and after traumatic head injury.[18].

Choline deficiency after cardiac arrest in the phase of the postresuscitation syndrome might be different. During the reperfusion syndrome after global hypoxia following cardiac arrest one can speculate that ongoing tissue damage and tissue recovery are potentially modified by mild therapeutic hypothermia. Although it is known that choline will be released initially due to tissue hypoxia, a decrease of choline to subnormal levels several hours after global hypoxia could possibly indicate increased uptake reflecting tissue repair and membrane synthesis.

Experimental data show that a combination of choline (administered in a bound form such as citicoline) and hypothermia is more effective than citicoline used alone in ameliorating cerebral damage after transient focal ischaemia.[19] The approach of choline substitution has only been evaluated in clinical trials with focal ischaemia (intracranial haemorrhage and stroke) using citicoline which is a choline metabolite and a precursor of phosphatidylcholine that is involved in cell membrane synthesis.[6] The results are contradictory so far. The ICTUS trial, a double-blinded, randomized, placebo-controlled, multi-center trial, did not find a benefit for choline substitution in patients with moderate-to-severe ischaemic stroke.[5] The pathophysiology of choline has been reviewed before in detail.[16] Secades et al. evaluated the effect of citicoline in patients with acute primary supratentorial hemispheric cerebral haemorrhage in a placebo-controlled trial revealing the safety of administered citicolin to humans.[20] The citicoline brain injury treatment (COBRIT) trial in patients with traumatic brain injury, a randomized, double-blind, placebo-controlled, multi-center trial, has investigated the effect of 90 days of citicoline treatment on functional outcome in patients with complicated mild, moderate, and severe traumatic brain injury.[21] Patients received placebo or citicoline 1000 mg twice a day, and the endpoint was a combination of different functional outcome tests. The published final results showed no improvement in functional and cognitive status between the randomized groups.[22] This negative results in patients after ischaemic stroke and traumatic brain injury underline the difficulty in comparing focal ischaemia with global hypoxia after cardiac arrest as stated before.

The detailed temporal profile of plasma free choline after cardiac arrest and hypothermia of our pilot trial carries important information for clinical trials investigating the potential of choline supplementation after cardiac arrest. Our data also inform on the relevant time points at which plasma choline determination should be performed to assess the efficacy of supplementation to restore blood choline levels. However, the prognostic implications of low plasma choline levels for patient outcome and possible beneficial role of choline supplementation in such patients remains speculative at this time. A possible choline deficiency during cell membrane regeneration and repair after cardiac arrest requires further investigation, as choline is essentially needed for cell membrane synthesis.[18], [21], [23] However low choline levels have been associated with impairments in cognitive development in other settings.[23] Supportive treatment of choline deficiency after cardiac arrest might be a novel approach in survivors after cardiac arrest to improve outcome. Choline is available for intravenous and oral substitution. The importance of correcting choline deficiency and adding choline to parenteral nutrition formulations has been recently outlined by the Amercian Society of Parenteral Nutrition but such recommendations are based on different patient groups and are currently not valid for other specific settings such as cardiac arrest.[24], [25].

## Conclusion

Plasma free choline levels were elevated immediately after resuscitation and decreased significantly to subnormal levels within 48–72 hours in hypothermia treated patients after cardiac arrest. During day two to five, we observed a sustained decrease of plasma choline. Subsequently concentrations recovered to normal levels at approximately six days after resuscitation. Choline deficiency during the reperfusion syndrome after global hypoxia might also indicate increased uptake reflecting tissue repair and membrane synthesis. If early choline deficiency after resuscitated cardiac arrest is a relevant pathophysiological mechanism, choline supplementation could represent a novel therapeutic strategy to improve neurological outcome in addition to the current standard of care.

## Key Messages

- Patients after cardiac arrest have initially elevated plasma choline levels on admission followed by a significant decrease to subnormal levels after 48–72 hours and a recovery within six days.
- Choline supplementation after cardiac arrest might be a novel therapeutic strategy to improve neurological outcome.

## References

[1] Bruhl A, Hafner G, Loffelholz K (2004) Release of choline in the isolated heart, an indicator of ischemic phospholipid degradation and its protection by ischemic preconditioning: no evidence for a role of phospholipase D. Life Sci 75: 1609–1620. 15261765 10.1016/j.lfs.2004.03.019

[2] Klein J, Holler T, Cappel E, Koppen A, Loffelholz K (1993) Release of choline from rat brain under hypoxia: contribution from phospholipase A2 but not from phospholipase D. Brain Res 630: 337–340. 8118702 10.1016/0006-8993(93)90674-c

[3] Korth U, Krieter H, Denz C, Janke C, Ellinger K, et al (2003) Intestinal ischaemia during cardiac arrest and resuscitation: comparative analysis of extracellular metabolites by microdialysis. Resuscitation 58: 209–217. 12909384 10.1016/s0300-9572(03)00119-9

[4] Zeisel SH (1985) Formation of unesterified choline by rat brain. Biochim Biophys Acta 835: 331–343. 4005286 10.1016/0005-2760(85)90289-9

[5] Davalos A, Alvarez-Sabin J, Castillo J, Diez-Tejedor E, Ferro J, et al (2012) Citicoline in the treatment of acute ischaemic stroke: an international, randomised, multicentre, placebo-controlled study (ICTUS trial). Lancet 380: 349–357. 22691567 10.1016/S0140-6736(12)60813-7

[6] Saver JL (2008) Citicoline: update on a promising and widely available agent for neuroprotection and neurorepair. Rev Neurol Dis 5: 167–177. 19122569

[7] Konstantinova SV, Tell GS, Vollset SE, Nygard O, Bleie O, et al (2008) Divergent associations of plasma choline and betaine with components of metabolic syndrome in middle age and elderly men and women. J Nutr 138: 914–920. 18424601 10.1093/jn/138.5.914

[8] Savendahl L, Mar MH, Underwood LE, Zeisel SH (1997) Prolonged fasting in humans results in diminished plasma choline concentrations but does not cause liver dysfunction. Am J Clin Nutr 66: 622–625. 9280183 10.1093/ajcn/66.3.622

[9] Bouwes A, Binnekade JM, Verbaan BW, Zandbergen EG, Koelman JH, et al (2012) Predictive value of neurological examination for early cortical responses to somatosensory evoked potentials in patients with postanoxic coma. J Neurol 259: 537–541. 21887511 10.1007/s00415-011-6224-5 PMC3296032

[10] Samaniego EA, Mlynash M, Caulfield AF, Eyngorn I, Wijman CA (2011) Sedation confounds outcome prediction in cardiac arrest survivors treated with hypothermia. Neurocrit Care 15: 113–119. 20680517 10.1007/s12028-010-9412-8 PMC3153345

[11] Mortberg E, Zetterberg H, Nordmark J, Blennow K, Rosengren L, et al (2011) S-100B is superior to NSE, BDNF and GFAP in predicting outcome of resuscitation from cardiac arrest with hypothermia treatment. Resuscitation 82: 26–31. 21071131 10.1016/j.resuscitation.2010.10.011

[12] Roivainen A, Forsback S, Gronroos T, Lehikoinen P, Kahkonen M, et al (2000) Blood metabolism of [methyl-11C]choline; implications for in vivo imaging with positron emission tomography. Eur J Nucl Med 27: 25–32. 10654143 10.1007/pl00006658

[13] Holm PI, Ueland PM, Kvalheim G, Lien EA (2003) Determination of choline, betaine, and dimethylglycine in plasma by a high-throughput method based on normal-phase chromatography-tandem mass spectrometry. Clin Chem 49: 286–294. 12560353 10.1373/49.2.286

[14] Leleiko RM, Vaccari CS, Sola S, Merchant N, Nagamia SH, et al (2009) Usefulness of elevations in serum choline and free F2)-isoprostane to predict 30-day cardiovascular outcomes in patients with acute coronary syndrome. Am J Cardiol 104: 638–643. 19699337 10.1016/j.amjcard.2009.04.047

[15] Danne O, Lueders C, Storm C, Frei U, Mockel M (2007) Whole blood choline and plasma choline in acute coronary syndromes: prognostic and pathophysiological implications. Clin Chim Acta 383: 103–109. 17553478 10.1016/j.cca.2007.05.001

[16] Danne O, Mockel M (2010) Choline in acute coronary syndrome: an emerging biomarker with implications for the integrated assessment of plaque vulnerability. Expert Rev Mol Diagn 10: 159–171. 20214535 10.1586/erm.10.2

[17] Ilcol YO, Uncu G, Goren S, Sayan E, Ulus IH (2004) Declines in serum free and bound choline concentrations in humans after three different types of major surgery. Clin Chem Lab Med 42: 1390–1395. 15576301 10.1515/CCLM.2004.259

[18] Ulus IH, Ozyurt G, Korfali E (1998) Decreased serum choline concentrations in humans after surgery, childbirth, and traumatic head injury. Neurochem Res 23: 727–732. 9566612 10.1023/a:1022455325657

[19] Sahin S, Alkan T, Temel SG, Tureyen K, Tolunay S, et al (2010) Effects of citicoline used alone and in combination with mild hypothermia on apoptosis induced by focal cerebral ischemia in rats. J Clin Neurosci 17: 227–231. 20036128 10.1016/j.jocn.2009.05.016

[20] Secades JJ, Alvarez-Sabin J, Rubio F, Lozano R, Davalos A, et al (2006) Citicoline in intracerebral haemorrhage: a double-blind, randomized, placebo-controlled, multi-centre pilot study. Cerebrovasc Dis 21: 380–385. 16490951 10.1159/000091547

[21] Zafonte R, Friedewald WT, Lee SM, Levin B, Diaz-Arrastia R, et al (2009) The citicoline brain injury treatment (COBRIT) trial: design and methods. J Neurotrauma 26: 2207–2216. 19803786 10.1089/neu.2009.1015 PMC2824223

[22] Zafonte RD, Bagiella E, Ansel BM, Novack TA, Friedewald WT, et al (2012) Effect of citicoline on functional and cognitive status among patients with traumatic brain injury: Citicoline Brain Injury Treatment Trial (COBRIT). JAMA 308: 1993–2000. 23168823 10.1001/jama.2012.13256

[23] Wu BT, Dyer RA, King DJ, Richardson KJ, Innis SM (2012) Early second trimester maternal plasma choline and betaine are related to measures of early cognitive development in term infants. PLoS One 7: e43448. 22916264 10.1371/journal.pone.0043448 PMC3423345

[24] Vanek VW, Borum P, Buchman A, Fessler TA, Howard L, et al (2012) A.S.P.E.N. position paper: recommendations for changes in commercially available parenteral multivitamin and multi-trace element products. Nutr Clin Pract 27: 440–491. 22730042 10.1177/0884533612446706

[25] Buchman AL (2009) The addition of choline to parenteral nutrition. Gastroenterology 137: S119–128. 19874943 10.1053/j.gastro.2009.08.010

