# Correction: Serial Plasma Choline Measurements after Cardiac Arrest in Patients Undergoing Mild Therapeutic Hypothermia: A Prospective Observational Pilot Trial

**DOI:** 10.1371/annotation/c26f7f58-76fa-4a6c-88da-128ea463abd7

**Published:** 2013-10-04

**Authors:** Christian Storm, Oliver Danne, Per Magne Ueland, Christoph Leithner, Dietrich Hasper, Tim Schroeder

During the production process of this article, the Academic Editor's attribution below the Citation was removed from the manuscript PDF. It should read:

Editor: Toru Hosoda, Tokai University, Japan 

